# Winter wheat yield prediction using temporal fusion of multispectral imagery and LiDAR point clouds acquired from a ground-based phenotyping platform

**DOI:** 10.3389/fpls.2026.1953299

**Published:** 2026-10-14

**Authors:** Haodong Li, Yeping Zhu, Shengping Liu, Fenghuan Du, Yu Zhang, Yihan Jiang, Run Li, Cunchao Li, Shijuan Li, Huili Yue

**Affiliations:** 1 Agricultural Information Institute, Chinese Academy of Agricultural Sciences, Beijing, China; 2 Institute of Agricultural Information and Economics, Hebei Academy of Agriculture and Forestry Sciences, Shijiazhuang, China

**Keywords:** ground-based phenotyping platform, lidar point clouds, multispectral imagery, pre-maturity yield prediction, temporal fusion, winter wheat

## Abstract

Winter wheat yield reflects the combined development of canopy structure and physiological status, making multitemporal structural and spectral observations potentially valuable for pre-maturity prediction. We used a ground-based phenotyping platform to acquire five-band multispectral imagery at 14 growth stages and LiDAR point clouds at 10 growth stages. Both data streams were aligned to a common phenological axis and integrated in a temporal graph convolutional network (Temporal-GCN) that encoded canopy spectral responses, three-dimensional structural states, and signed changes between adjacent stages. A stagewise cutoff analysis evaluated yield prediction from erecting through maturity. Multitemporal multispectral trajectories captured SPAD dynamics, whereas LiDAR directly provided plant height and achieved high accuracy in LAI estimation, indicating complementary sensing responses. In three-fold cross-validation grouped by complete biological replicate, the fused model comprising VARI, EVI, NDRE, and seven point-cloud structural variables reached R^2^ = 0.883 at heading and its highest full-season cutoff point estimate at early grain filling (R^2^ = 0.903, RMSE = 25.52 kg mu^-1^, MAE = 20.84 kg mu^-1^). The corresponding current-stage-only model yielded R^2^ = 0.760. Paired bootstrap analysis indicated a cumulative-versus-current-stage improvement of ΔR^2^ = 0.143 (95% CI, 0.059–0.299) and an RMSE reduction of 14.69 kg mu^-1^ (95% CI, 7.28–22.02 kg mu^-1^). Performance remained similar at mid grain filling (R^2^ = 0.900), and the early-versus-mid difference was small (ΔR^2^ = 0.003; 95% CI, −0.015 to 0.025). A nested grouped sensitivity analysis for endpoint-pooling and regularization yielded R^2^ = 0.896. Early grain filling occurred approximately 27 d before harvest. These results show that multitemporal structural–spectral information can improve pre-maturity winter wheat yield prediction, while the identified prediction window should be interpreted within the present one-site, one-season experiment.

## 1 Introduction

Winter wheat yield develops through a continuous sequence of stand establishment, canopy expansion, flowering and seed set, and grain filling. Conventional field surveys generally characterize plant height, leaf greenness, or leaf area at individual time points. Repeated measurements are constrained by labor requirements, implementation costs, and destructive sampling for some traits. Advances in high-throughput phenotyping have enabled continuous, nondestructive observation of crop development (Furbank and Tester, 2011; Fiorani and Schurr, 2013; Cobb et al., 2013; Araus and Cairns, 2014). Field phenotyping platforms and low-altitude remote sensing have further increased the spatial coverage and temporal frequency of field observations (White et al., 2012; Walter et al., 2015; Sankaran et al., 2015). Crop phenotyping has consequently progressed from single-stage trait measurement toward multitemporal sensing that captures biologically meaningful growth dynamics and supports yield assessment before maturity (Tardieu et al., 2017; Maes and Steppe, 2019).

Multispectral sensing records crop-canopy reflectance in the visible, red-edge, and near-infrared regions. Vegetation indices derived from these bands can characterize canopy greenness, chlorophyll status, and green leaf area (Rouse et al., 1974; Huete, 1988; Gitelson et al., 2002; Huete et al., 2002; Haboudane et al., 2004; Gitelson et al., 2005; Yan et al., 2025). With the development of ground-based phenotyping platforms and uncrewed aerial systems, multispectral analysis has expanded from single-stage diagnosis to multitemporal monitoring of wheat nitrogen status, canopy senescence, and yield formation (Hassan et al., 2018; Jiang et al., 2022). LiDAR extends crop observation from two-dimensional spectral responses to three-dimensional canopy structure and supports quantitative assessment of plant height, canopy cover, aboveground biomass, leaf angle, and leaf area index (LAI) (Madec et al., 2017; Jimenez-Berni et al., 2018; Yang et al., 2023; Wang et al., 2024; Mulero et al., 2025). The two sensors operate through different measurement mechanisms. Multispectral imagery primarily records changes in canopy color and red-edge and near-infrared reflectance, whereas LiDAR point clouds retain information on canopy height, apparent volume, vertical return distributions, and structural complexity. The two modalities therefore emphasize spectral responses associated with canopy physiological status and three-dimensional canopy structure, respectively, and may provide complementary information.

This complementarity has encouraged a shift from single-source regression toward multimodal and multitemporal crop-yield modeling (van Klompenburg et al., 2020; Maimaitijiang et al., 2020; Li et al., 2022), and fusion of multispectral imagery and point clouds has also been used to characterize three-dimensional photosynthetic traits in wheat canopies (Gu et al., 2024). Increasing the number of sensors or variables, however, does not necessarily improve prediction. Multiple vegetation indices can encode similar canopy-greenness or red-edge signals, while point-cloud height percentiles, cover metrics, and volume variables are often strongly correlated. Direct concatenation of high-dimensional inputs can therefore add redundancy, noise, and estimation variance. Existing multitemporal yield-prediction studies also differ in sensor combinations, model structures, observation endpoints, and data-partitioning strategies (Cao et al., 2020; Sun et al., 2022; Tanabe et al., 2023; Yang et al., 2024; Ge et al., 2025; Xiong et al., 2026). Some studies have achieved useful predictions before or near flowering (Camenzind and Yu, 2024), but differences in model and validation design make it difficult to isolate the contributions of additional growth-stage information and multisource fusion. In particular, assigning records from different stages of the same plot to separate training and test sets can allow a model to exploit plot-specific differences rather than generalizable temporal patterns, resulting in optimistic performance estimates (Roberts et al., 2017). A controlled comparison should therefore limit input redundancy, keep the model and grouped validation scheme fixed, and expand the observation window sequentially. Such a design can quantify the incremental value of multisource fusion and historical observations and identify the growth stage at which the highest full-season prediction accuracy emerges.

The primary objective of this study was to evaluate pre-maturity winter wheat yield prediction using temporally integrated multispectral and LiDAR observations. The experiment included three winter wheat cultivars, four nitrogen rates, and three biological replicates, with multispectral imagery acquired at 14 growth stages and LiDAR point clouds at 10 stages. Plant height, relative leaf chlorophyll content (SPAD), and LAI were used as supporting phenotypes to characterize the structural and spectral information represented by the two sensors. We then selected low-redundancy spectral and structural features on the basis of correlation structure, training-fold relevance, and agronomic interpretation. Multispectral-only, point-cloud-only, and fused models were compared on a common phenological axis using the same Temporal-GCN architecture and grouped cross-validation scheme. Finally, the observation window was expanded one stage at a time, with each cutoff containing only measurements available by that stage. The analysis quantified the incremental contribution of multispectral information relative to a point-cloud structural baseline, the value of historical observations relative to current-stage-only inputs, and the stage at which high pre-maturity prediction accuracy emerged.

## 2 Materials and methods

### 2.1 Experimental design and data acquisition

The field experiment was conducted during the 2025–2026 winter wheat growing season at the Dahe Town experimental station in Luquan District, Shijiazhuang, Hebei Province, China (38.1261° N, 114.3899° E; approximately 75 m above sea level). The region has a warm-temperate continental monsoon climate, and the experimental soil is predominantly cinnamon soil. Meteorological and soil information was used only to characterize the experimental environment and was not supplied to the prediction models. Winter wheat was sown on 27 October 2025 and harvested on 8 June 2026. The sowing rate was 20 kg mu^-1^ (40 jin mu^-1^; 1 mu = 1/15 ha and 1 jin = 0.5 kg), with a row spacing of 15 cm. The cultivars were Malan No. 1, Lunxuan 45, and Jimai 765, and the four nitrogen rates were 0, 180, 240, and 300 kg N ha^-1^. Each cultivar-by-nitrogen combination had three biological replicates, producing 36 experimental plots of 2 m × 2 m. The four nitrogen treatments were arranged in strips, and each nitrogen strip contained all three cultivars and their three biological replicates; the experiment did not follow a randomized block design. Nitrogen was supplied as urea. Forty percent of the target nitrogen rate was applied 1 d before sowing, and 30% was applied on each of 17 March and 8 April 2026. All plots were irrigated once on 17 March, 8 April, and 7 May. Pest and disease control and all other field-management practices were consistent across plots except for nitrogen rate. The location, field layout, and plot identifiers are shown in **Figure 1**.

**Figure 1:**
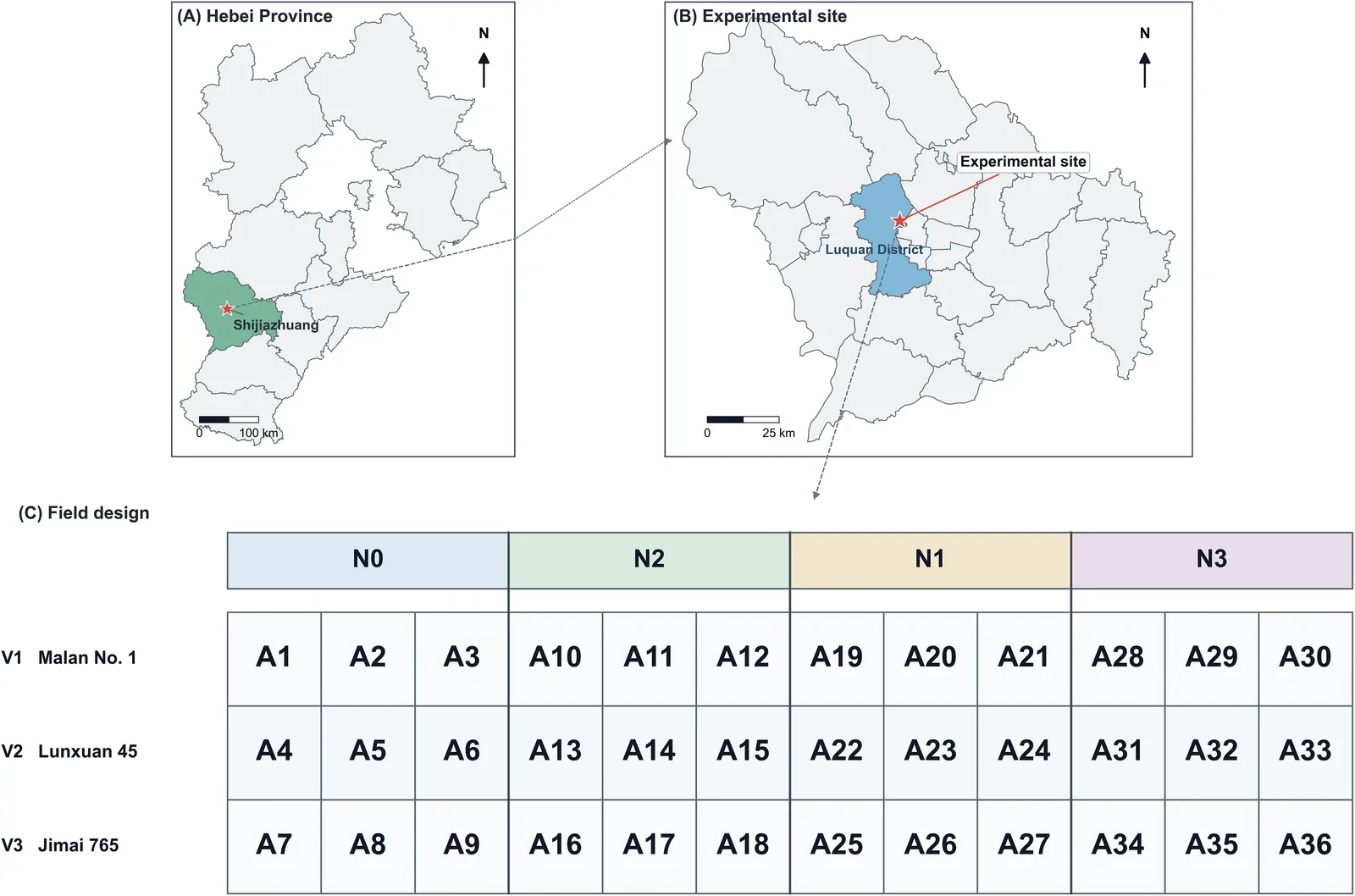
Experimental location and field design. **(A)** Location of Shijiazhuang in Hebei Province, China. **(B)** Location of the experimental site in Luquan District, Shijiazhuang. **(C)** Layout of the 36 field plots. V1–V3 denote Malan No. 1, Lunxuan 45, and Jimai 765, respectively; N0, N1, N2, and N3 denote nitrogen application rates of 0, 180, 240, and 300 kg N ha^-1^, respectively.

Plant height was measured on 10 plants in each plot, and their mean represented plot-level plant height. Relative leaf chlorophyll content was measured on the most recently fully expanded leaf of the main stem with a SPAD-502 chlorophyll meter. Ten SPAD readings were obtained for each plot at each stage and averaged. LAI was measured with an LAI-2200C canopy analyzer fitted with a 45° view cap to restrict the azimuthal field of view and reduce contributions from neighboring plots and other nontarget areas. Each measurement sequence comprised one above-canopy reference reading (A) and four below-canopy readings (B). Ten such sequences were completed in each plot, and the resulting ten LAI estimates were averaged to represent plot-level LAI. Sensor height, orientation, and operating procedure were kept consistent, and the interval between the A and B readings within a sequence was minimized to limit the effect of short-term changes in illumination. To assess the reliability of instrument-derived LAI, destructive measurements were conducted concurrently at five representative growth stages, yielding 180 paired instrument and manual observations from the 36 plots. At maturity, a 1 m × 1 m area in each plot was harvested. Grain was threshed, air-dried until its mass stabilized, weighed, and converted to yield per unit area (kg mu^-1^). No correction to a standard grain moisture content was applied; yield is therefore reported on an air-dry basis.

Manual LAI was determined by destructive sampling and the specific leaf area method. A 20-cm segment spanning two rows was removed from each plot and transported to the laboratory, where plants were separated into stems, leaves, and spikes. Representative leaves were selected as reference leaves, and their one-sided leaf area was measured. The reference leaves and the remaining leaves were bagged separately, dried to constant mass, and weighed. Total leaf dry mass in the sampled segment was calculated as the sum of the two fractions. Specific leaf area was calculated from the area and dry mass of the reference leaves and multiplied by total leaf dry mass to estimate total leaf area in the sampled segment. Manual LAI was then calculated as the ratio of total one-sided leaf area to the corresponding ground area. Instrument-derived and manual LAI observations were paired by plot and growth stage. Destructive-sampling areas were spatially separated from subsequent sensor-observation areas and from the 1 m × 1 m maturity-harvest area to prevent interference with later measurements.

(1) $$SLA=\frac{A_{s}}{W_{s}}$$

(2) $$A_{t}=(\frac{A_{s}}{W_{s}})W_{t}$$

(3) $$LAI=\frac{A_{t}}{S_{g}}=\frac{A_{s}W_{t}}{W_{s}S_{g}}$$

In (Equations 1-3), SLA is the specific leaf area of the reference leaves (cm^2^ g^-1^). A_s_ and W_s_ are the measured one-sided area (cm^2^) and oven-dry mass (g), respectively, of those leaves. W_t_ is the oven-dry mass of all leaves in the sampled segment, and A_t_ is the total leaf area estimated from SLA and W_t_ (cm^2^). S_g is the ground area corresponding to the 20-cm two-row segment (cm^2^), and LAI is the dimensionless one-sided leaf area per unit ground area.

Canopy phenotypes were acquired at fixed positions with the LiCrop ground-based crop phenotyping platform developed by Beijing GreenValley Technology Co., Ltd. (Beijing, China). The platform and the overall analysis workflow are shown in **Figure 2**. LiCrop integrated a MicaSense RedEdge-MX five-band multispectral camera, a FARO P70A three-dimensional laser scanner, and an RGB camera, enabling multispectral imagery, three-dimensional point clouds, and visible-light imagery to be collected within the same workflow. The platform was positioned over the center of each 2 m × 2 m plot for stationary acquisition, with the sensors approximately 2.5 m above the plot plane. No multi-station point-cloud registration was performed. Multispectral data characterized canopy spectral properties and spectral changes associated with physiological status, whereas point clouds described canopy height, cover, and vertical structure. RGB imagery documented canopy conditions and supported data-quality assessment. Each acquisition campaign began after all three cultivars had reached the corresponding growth stage, and all observations in that campaign were assigned the same stage label. At each stage, all 36 plots were measured on the same day between 10:00 and 14:00 with consistent device settings, platform height, and acquisition order. This interval was centered near local solar noon to reduce extreme shadow differences associated with low solar elevation in the early morning or late afternoon. Radiometric calibration, restriction to the common valid image footprint, and vegetation-pixel screening were subsequently applied to improve comparability among plots within a campaign (Section 2.2). Multispectral imagery covered 14 growth stages, and LiDAR point clouds covered 10 of these stages. Sensor observations and manual measurements were managed with a composite plot-by-stage identifier to ensure correspondence among multispectral imagery, LiDAR point clouds, RGB imagery, and ground phenotypes. The complete acquisition schedule is given in **Table 1**.

**Figure 2:**
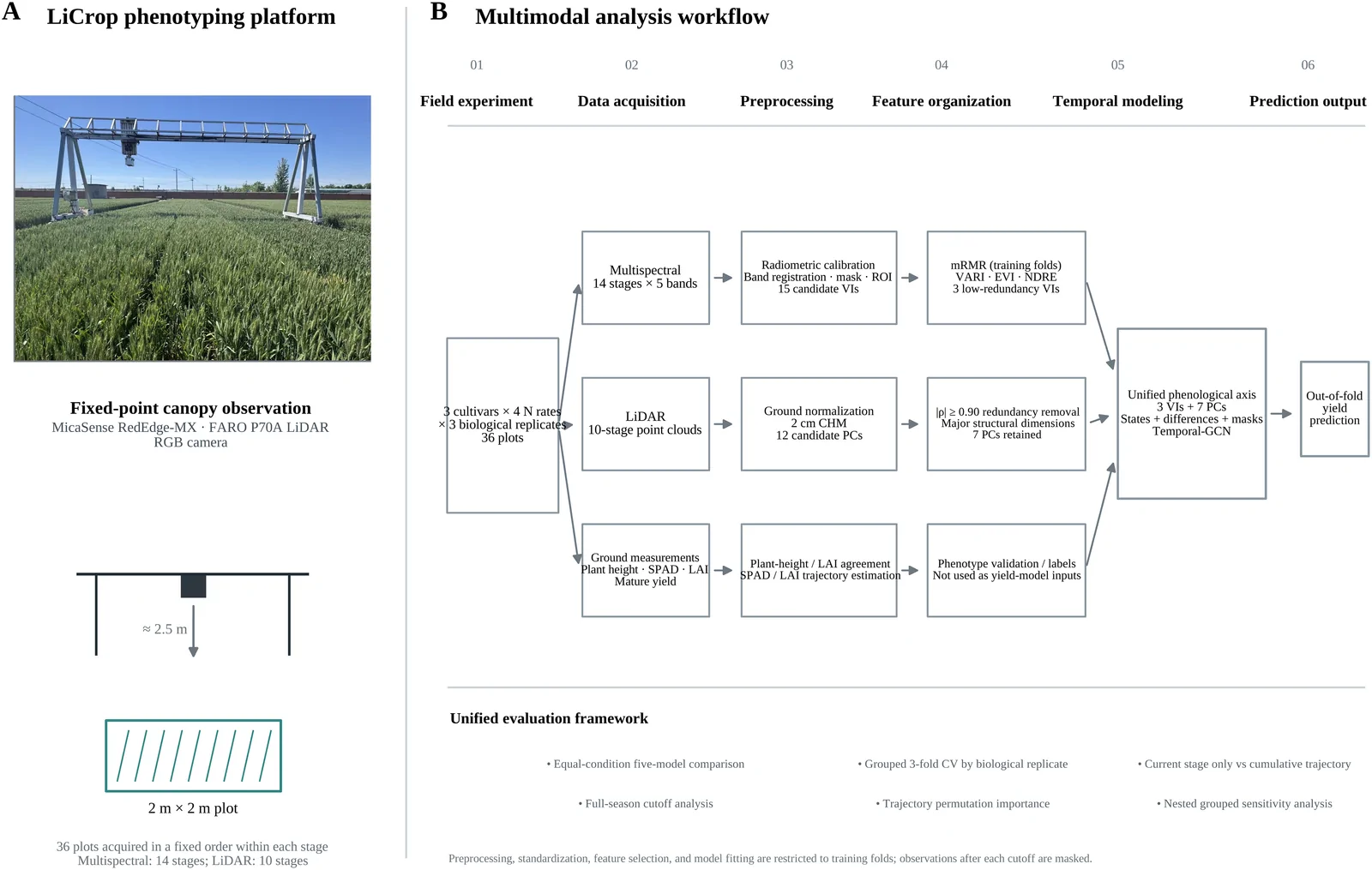
LiCrop ground-based phenotyping platform and multimodal analysis workflow. **(A)** LiCrop platform and fixed-point canopy observation geometry. **(B)** Workflow linking the field experiment, multimodal data acquisition, preprocessing, feature organization, temporal modeling, out-of-fold yield prediction, and the unified evaluation framework.

**Table 1:** Experimental design, dataset size, and acquisition schedule.

A. Experimental design and dataset size					
Data component		Acquisition design and sample size		Data content	
Experimental design		3 cultivars × 4 nitrogen rates × 3 biological replicates		36 plots of 2 m × 2 m	
Multispectral imagery		14 stages × 36 plots × 5 bands		15 vegetation indices	
Three-dimensional point clouds		10 stages × 36 plots		12 candidate structural variables	
Ground phenotypes		9 common stages × 36 plots		324 observations each for plant height, SPAD, and LAI	
LAI validation		5 stages × 36 plots		180 paired manual and LAI-2200C measurements	
Grouped cross-validation		One complete biological replicate held out per fold		24 training plots and 12 held-out plots per fold	

B. Sensor acquisition schedule (yield harvest: 8 June 2026)					
Growth stage/date	Multispectral	LiDAR	Growth stage/date	Multispectral	LiDAR
Emergence, 19 Nov 2025	•	—	Late booting, 23 Apr 2026	•	•
Pre-winter, 10 Dec 2025	•	—	Heading, 29 Apr 2026	•	•
Overwintering, 27 Jan 2026	•	—	Flowering, 7 May 2026	•	•
Green-up, 11 Mar 2026	•	—	Early grain filling, 12 May 2026	•	•
Erecting, 25 Mar 2026	•	•	Mid grain filling, 21 May 2026	•	•
Jointing, 1 Apr 2026	•	•	Late grain filling, 28 May 2026	•	•
Booting, 14 Apr 2026	•	•	Maturity, 4 Jun 2026	•	•

Filled circles indicate that data were acquired; em dashes indicate no acquisition. Yield was measured at harvest on 8 June 2026.

### 2.2 Data preprocessing

The multispectral imagery comprised blue, green, red, red-edge, and near-infrared bands. A raw RedEdge-MX single-band image contained 1280 × 960 pixels, with a pixel size of 3.75 μm and a focal length of 5.4 mm. At an acquisition height of approximately 2.5 m, the ground sampling distance was approximately 1.7 mm pixel^-1^. Preprocessing proceeded through dark-current and noise correction, lens-distortion correction, vignetting correction, radiometric calibration, five-band registration, and plot-boundary cropping. A calibrated reflectance panel was imaged before and after each acquisition. A linear relationship between image digital numbers and nominal panel reflectance was established separately for each band. A common calibration coefficient was used within an acquisition batch when illumination was stable, whereas batches or acquisitions with pronounced illumination changes were calibrated separately to reduce differences caused by solar irradiance and camera response. The green band served as the geometric reference for registration, and phase correlation was used to estimate the translation of each remaining band relative to the green band. When displacement estimation was unstable for an individual image, it was constrained using the robust displacement estimate for the corresponding acquisition batch or growth stage. This procedure produced spatially aligned, plot-level reflectance imagery. A vegetation mask was generated from the NDVI image for each growth stage using an adaptive Otsu threshold, with plausible bounds imposed on anomalous threshold estimates. Only pixels within the common valid footprint of all five bands were retained. Plot-level vegetation indices were calculated as the mean of vegetation-pixel values between the 2nd and 98th percentiles to reduce the influence of extreme pixels. Multispectral regions of interest (ROIs) were defined from the field boundaries of the 2 m × 2 m plots and were moderately inset to reduce contributions from neighboring treatments and mixed boundary pixels.

Vegetation indices used in the subsequent correlation analysis were selected according to three criteria: they could be calculated from the five camera bands, had an established application in crop multispectral sensing, and represented distinct canopy spectral responses. Definitions were based on the original index publications and subsequent reviews (Rouse et al., 1974; Huete, 1988; Qi et al., 1994; Goel and Qin, 1994; Rondeaux et al., 1996; Louhaichi et al., 2001; Gitelson et al., 2002; Huete et al., 2002; Haboudane et al., 2004; Gitelson et al., 2005; Jiang et al., 2008; Yan et al., 2025). After radiometric calibration, band registration, and vegetation masking, we calculated 15 candidate indices: NDVI, GNDVI, NDRE, EVI, EVI2, SAVI, OSAVI, VARI, GLI, MSAVI, CIgreen, CIrededge, RVI, DVI, and NLI. VegetationFraction was used only to assess mask quality and was not treated as a candidate model predictor (**Table 2**).

**Table 2:** Multispectral vegetation indices.

Index	Formula	Principal phenotypic interpretation
NDVI	(NIR − R)/(NIR + R)	Canopy greenness
OSAVI	1.16(NIR − R)/(NIR + R + 0.16)	Adjustment for soil background
SAVI	1.5(NIR − R)/(NIR + R + 0.5)	Adjustment for soil background
EVI	2.5(NIR − R)/(NIR + 6R − 7.5B + 1)	Relative canopy spectral response
EVI2	2.5(NIR − R)/(NIR + 2.4R + 1)	Two-band enhancement
GNDVI	(NIR − G)/(NIR + G)	Chlorophyll and nitrogen status
CIrededge	NIR/RE − 1	Red-edge chlorophyll response
MSAVI	[2NIR + 1 − √((2NIR + 1)^2^ − 8(NIR − R))]/2	Adaptive soil adjustment
DVI	NIR − R	Difference in vegetation amount
RVI	NIR/R	Ratio-based greenness
CIgreen	NIR/G − 1	Green-band chlorophyll response
VARI	(G − R)/(G + R − B)	Visible-band greenness
GLI	(2G − R − B)/(2G + R + B)	Proportion of green foliage
NLI	(NIR^2^ − R)/(NIR^2^ + R)	Nonlinear response
NDRE	(NIR − RE)/(NIR + RE)	Red-edge and chlorophyll status

B, G, R, RE, and NIR denote blue, green, red, red-edge, and near-infrared reflectance, respectively.

Raw LAS point clouds were standardized in coordinate orientation, cropped by plot, and ground-normalized to obtain height above ground, h_i_. The point-cloud ROI followed the actual 2 m × 2 m field boundary of each plot and had no fixed inset buffer. For each plot, ground elevation was estimated from the 0.5th percentile of the lower-layer return-elevation distribution, and normalized ground height was set to h = 0. This robust reference reduced the influence of extreme low outliers on ground estimation. Spatial completeness, valid point count, and the height distribution of each plot were checked before feature extraction. A vertically stratified voxel-downsampling strategy was used to reduce redundant returns in high-density canopy regions while preferentially retaining the sparser returns from the middle and lower canopy. All plots within a growth stage were processed with the same rules. Each plot was processed independently, without cross-plot or multi-station point-cloud registration. All subsequent structural variables were calculated from point clouds after ground normalization and stratified downsampling. A canopy height model (CHM) was then constructed on a 2-cm grid. The maximum height above ground in grid cell j was denoted H_t_, and cells without returns were assigned a value of 0. Point-cloud-derived plant height was represented by the 99.5th percentile of height above ground. Canopy cover at thresholds of 5 and 20 cm was calculated as the proportion of CHM grid area at or above the corresponding threshold. Apparent canopy volume was obtained by integrating grid height. Middle- and upper-layer point ratios described the allocation of returns among relative-height intervals, and vertical entropy quantified the dispersion of returns across 20 equal-height strata. The categories, units, and phenotypic interpretations of the 12 candidate structural variables are given in **Table 3**; the principal calculations are shown in (Equations 4-8).

**Table 3:** Point-cloud structural variables.

Category	Variable	Unit	Phenotypic interpretation
Height	H99.5, H90, H75, H50	cm	Canopy top and vertical strata
Dispersion	SDH, CVH	cm; dimensionless	Vertical dispersion and relative variation
Cover	CanopyCover_5cm, CanopyCover_20cm	%	Area cover above different height thresholds
Volume	CHM_Volume	m³	Integral of the canopy height model
Vertical distribution	UpperPointRatio, MidPointRatio	dimensionless	Proportions of upper- and middle-layer points
Complexity	VerticalEntropy	dimensionless	Complexity among vertical strata

H99.5, H90, H75, and H50 are height percentiles; SDH and CVH are the standard deviation and coefficient of variation of point height, respectively; CHM denotes canopy height model. The table lists all 12 candidate point-cloud structural variables.

(4) $$H_{99.5}=Q_{0.995}(h_{i},i=1,\ldots ,N)$$

(5) $$C_{\tau }=\frac{1}{M}\sum_{j=1}^{M}I(H_{j}\geq \tau )\times 100\%,\tau \in \{0.05,0.20\}\text{m}$$

(6) $$V_{\text{CHM}}=g^{2}\sum_{j=1}^{M}H_{j},g=0.02\text{m}$$

(7) $$\begin{array}{c} R_{\text{mid}}=\frac{1}{N}\sum_{i=1}^{N}I(0.30H_{99.5}\leq h_{i}<0.70H_{99.5})\\ R_{\text{upper}}=\frac{1}{N}\sum_{i=1}^{N}I(h_{i}\geq 0.70H_{99.5}) \end{array}$$

(8) $$E=-\frac{\sum_{k=1}^{20}p_{k}\text{ln}(p_{k})}{\text{ln}(20)}$$

In (Equations 4-8), h_i_ is the height above ground of point i (m), N is the number of points in a plot, Q_0.995_ is the 99.5th percentile, and I(·) is the indicator function. H_t_ is the maximum height above ground in the jth 2-cm CHM cell, M is the number of cells, τ is 0.05 or 0.20 m, and g is the cell width (0.02 m). The proportion of points in height stratum k is p_k_, with 0 ln 0 defined as 0. H99.5 is described as point-cloud-derived plant height in the text, whereas figures, tables, and equations retain the parameter name H99.5. Representative multitemporal multispectral and point-cloud observations are shown in **Figure 3**.

**Figure 3:**
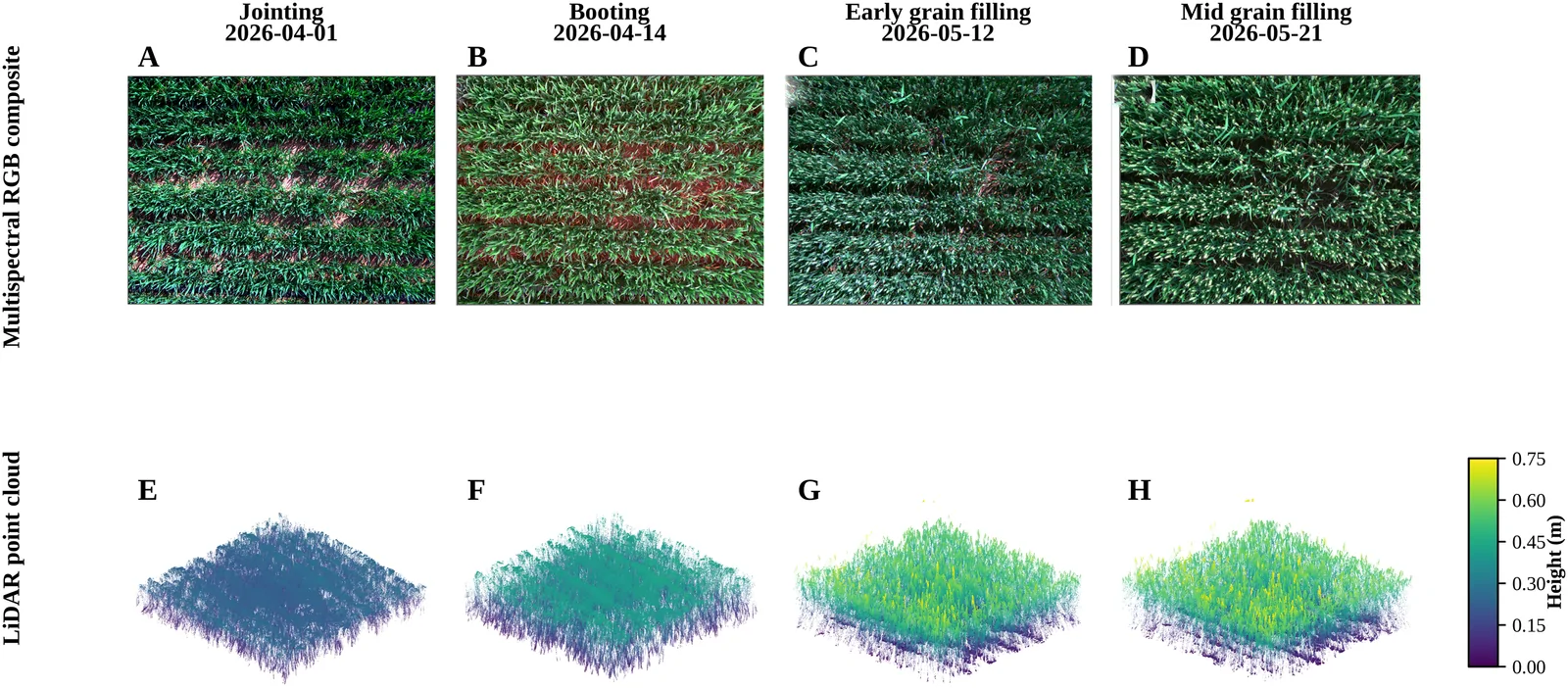
Representative multitemporal multimodal observations from plot 2. **(A–D)** Multispectral RGB composites acquired at jointing, booting, early grain filling, and mid grain filling. **(E–H)** Corresponding LiDAR point clouds, with color indicating height above ground. The RGB composites were generated from the registered red, green, and blue bands. All valid points were displayed using a common viewing direction and height scale. These renderings were used only for visualization and were not used for feature calculation or model fitting.

### 2.3 Multisource temporal fusion and stagewise cutoff analysis

Multisource temporal information was organized around three considerations: differences in sensor response, control of variable redundancy, and the temporal boundary of the available observations. Manually measured plant height, relative leaf chlorophyll content (SPAD), and LAI were used to assess how the two sensor types represented canopy three-dimensional structure and associated spectral responses. Point-cloud-derived plant height was represented by H99.5 and compared with manual plant height. SPAD and LAI were estimated using both stage-specific observations and full-season trajectories to compare how multispectral responses and point-cloud structural trajectories represented different phenotypic traits. Because plant height was measured directly from the point clouds, no multispectral or fused plant-height model was constructed. Estimated SPAD and LAI were used only to interpret differences in sensor response and were not supplied to the yield model, thereby avoiding propagation of intermediate phenotype-estimation error.

The 15 vegetation indices were ranked using the minimum redundancy–maximum relevance (mRMR) criterion (Peng et al., 2005). Because the model input was a multitemporal sequence, mutual information between each index and final yield was estimated separately at the eight model stages from green-up through early grain filling and then averaged across stages to quantify relevance. Redundancy between candidate indices was likewise defined as their mean mutual information across the eight stages. Difference-form mRMR was applied sequentially so that each added variable retained high relevance to yield while minimizing information already represented by the selected variables. Mutual information between continuous variables was estimated with k = 3 nearest neighbors. To examine selection stability, mRMR was run independently within the 24 training plots corresponding to each held-out biological replicate. The three training-fold ranks were averaged to produce a cross-fold summary; ties in mean rank were resolved by the higher mean target mutual information across folds. VARI, NDRE, and EVI occupied the first three positions in this summary and constituted the final three-index set. Input-size sensitivity followed the same summary ranking. The four-index set added CIgreen; the eight-index set further added CIrededge, DVI, RVI, and NLI; and the 15-index set included all candidates. For the point-cloud variables, pairs with |ρ| ≥ 0.90 were treated as highly redundant. Seven representative variables were retained to cover height, relative height variation, cover, apparent volume, vertical return allocation, and structural complexity: point-cloud-derived plant height, CVH, CanopyCover_5cm, CHM_Volume, UpperPointRatio, MidPointRatio, and VerticalEntropy. These screening outcomes are treated as within-dataset feature-development choices rather than as an independently validated optimal subset.

The 14 multispectral observations and 10 point-cloud observations were mapped to a common axis according to their observed growth stages. The multispectral sequence began at green-up, whereas point-cloud acquisition began at erecting. Nodes without point-cloud observations were identified by a sensor-availability mask; neither adjacent-stage nor later-stage observations were used for imputation. At every temporal node, the model received the current state of each variable and its signed difference from the preceding stage:

(9) $$\text{Δ}x_{t}=x_{t}-x_{t-1}$$

In (Equation 9), x_t_ is the observed value of a variable at growth stage t, and Δx_t_ represents the direction and magnitude of its change between adjacent stages. State values describe the canopy phenotype at a particular stage, whereas difference terms encode temporal change. If a valid observation was absent at either of two adjacent stages, the corresponding difference was left missing. State and difference variables were standardized using statistics fitted exclusively to the relevant training fold. Missing entries were then set to zero, and the sensor-availability masks identified whether a node contained an actual observation, preventing structural nonacquisition from being interpreted as a true zero. No observations from neighboring or later stages were used to fill missing values after quality control.

The aligned sequences were encoded with Temporal-GCN. Each phenological stage was represented as a graph node, with node features comprising spectral and structural state values, signed adjacent-stage differences, and sensor-availability masks. Self-loops and bidirectional edges between adjacent stages represented continuity in canopy development, and the same normalized adjacency matrix was used in both graph-convolution layers. The fused model contained 22 node-level inputs: 10 state variables, 10 corresponding differences, and two modality-availability masks. A 22→10 linear embedding followed by a rectified linear unit (ReLU) and dropout transformed each node input. Two graph-convolution layers then encoded the sequence, first from 10 to 10 features and then from 10 to 8 features. To retain both the full trajectory and the current endpoint, endpoint-aware readout combined the global mean of all encoded nodes with the representation of the current cutoff node. The main model assigned weights of 0.70 and 0.30 to the global-mean and current-node representations, respectively, and an 8→1 linear layer produced the yield prediction. Multispectral-only, point-cloud-only, and fused routes used the same node organization and Temporal-GCN architecture and differed only in the input variables visible to the model. Each single-source route retained only the availability mask for its own sensor, whereas the fused route retained both masks, preventing the availability of an unseen modality from entering a single-source model. The overall workflow is shown in **Figure 2B**, and the network architecture is shown in **Figure 4**.

**Figure 4:**
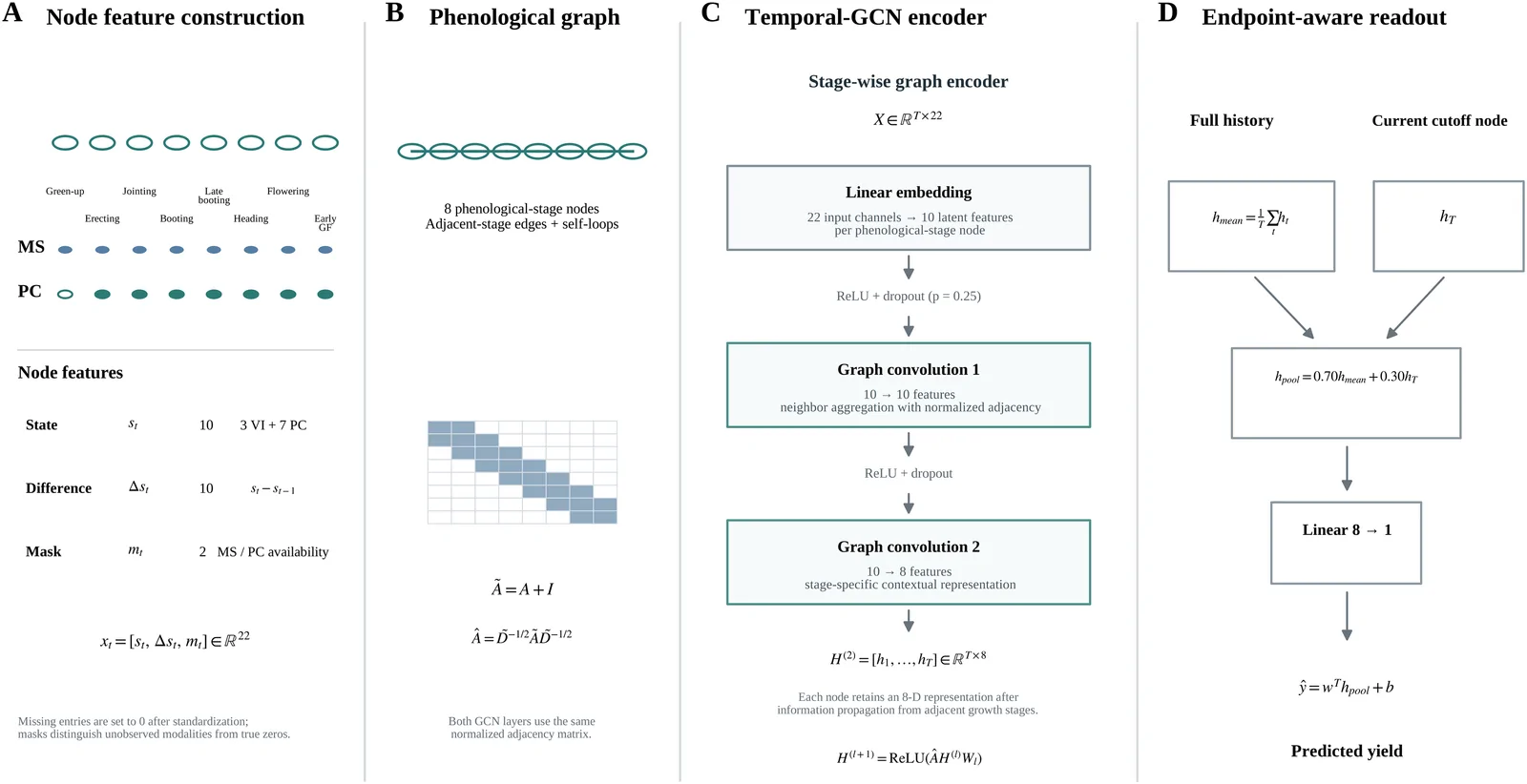
Architecture of the Temporal-GCN. **(A)** Node features comprise stage-specific state values, signed differences between adjacent stages, and multispectral and point-cloud availability masks. **(B)** The phenological graph contains eight growth-stage nodes, edges between adjacent stages, and self-loops. **(C)** A 22-dimensional node input is mapped through a linear embedding and two graph-convolution layers to an eight-dimensional representation for each node. **(D)** Endpoint-aware readout combines the full-history mean representation and the current cutoff-node representation with fixed weights of 0.70 and 0.30, respectively, before a linear layer predicts yield.

To evaluate pre-maturity yield prediction across the season, the stagewise cutoff analysis advanced from erecting through maturity by adding one new growth stage at each step and masking all observations after the cutoff. Every cutoff used the same final feature set, Temporal-GCN architecture, three-fold partition by complete biological replicate, and fixed-seed rule. Model structure and feature-selection rules were not redefined for individual cutoffs. Accordingly, the stagewise cutoff analysis was a fixed-protocol comparison rather than a cutoff-specific model search: no feature re-screening, architecture changes, or hyperparameter re-optimization were performed for individual cutoffs. A current-stage-only comparator was also fitted with the same training configuration but received only the state values and sensor-availability masks at the target stage. This comparison was used to evaluate the incremental value of historical trajectories. The resulting framework linked sensor-response validation, low-redundancy feature fusion, asynchronous temporal encoding, and full-season cutoff evaluation in a common analysis chain. Network parameters, training procedures, and evaluation rules are described in Section 2.4.

### 2.4 Model construction and evaluation

Candidate regressors for SPAD and LAI included partial least-squares regression, radial-basis-function support vector regression (RBF-SVR), random forest, and extremely randomized trees (ExtraTrees) (Cortes and Vapnik, 1995; Wold et al., 2001; Breiman, 2001; Geurts et al., 2006). Full-season trajectory estimation used multioutput models to estimate trait values at the nine growth stages common to both sensors. Multispectral ExtraTrees was ultimately used for SPAD, and point-cloud ExtraTrees was used for LAI. Yield prediction compared multisource partial least-squares regression (MS-PLS), temporal support vector regression (temporal SVR), a lightweight temporal convolutional network (TCN), a lightweight long short-term memory (LSTM) network, and Temporal-GCN (Cortes and Vapnik, 1995; Hochreiter and Schmidhuber, 1997; Wold et al., 2001; Kipf and Welling, 2017; Bai et al., 2018). All five models used the same three vegetation indices and seven point-cloud variables, the same eight phenological nodes from green-up through early grain filling, and the same three-fold partition by complete biological replicate. For the three neural temporal models, each 22-dimensional node input was first transformed by a 22→10 linear embedding followed by ReLU activation and dropout before entering the model-specific temporal encoder. MS-PLS and temporal SVR flattened the common 8 × 22 temporal tensor into 176 inputs; missing-value imputation, scaling, and feature selection were fitted only within the corresponding training fold. MS-PLS used four latent variables. Temporal SVR used RobustScaler, selected the top 30 features by an F test fitted within the training fold, and applied an RBF kernel with C = 100, ϵ = 8, and γ = 0.003. The TCN used two one-dimensional convolutional layers (kernel size = 3; 10→10→8) followed by temporal mean pooling. The LSTM used a single recurrent layer with a hidden dimension of 10 and retained the final hidden state. For the common five-model comparison, dropout, weight decay, and learning rate were 0.10, 0.02, and 0.007 for the TCN and 0.15, 0.04, and 0.007 for the LSTM, respectively. Temporal-GCN used the 22→10 linear embedding and the 10→10 and 10→8 graph-convolution layers described above. Its main configuration used dropout = 0.25, learning rate = 0.006, weight decay = 0.08, and 420 training epochs. All three neural temporal models were optimized with AdamW and mean squared error loss, and predictions were averaged across five fixed random seeds. After the five-model comparison, Temporal-GCN was fixed for the single-source, fused, input-size, and stagewise cutoff analyses. The current-stage-only comparator used the same training configuration but received only target-stage state values and sensor masks.

All plots were evaluated by three-fold grouped cross-validation defined by complete biological replicate. In each fold, 24 plots from two replicates were used for training, and the 12 plots in the remaining replicate formed the held-out fold. All growth-stage records from a plot remained on the same side of the split, preventing temporal records from crossing folds. Missing-value imputation, standardization, feature selection, and model fitting used only the corresponding training-fold data. The main three-fold analysis was designed to characterize prediction stability across independent biological replicates within the cultivar-by-nitrogen combinations observed in this experiment rather than to estimate transfer to a completely unseen management level. Because endpoint-aware pooling and regularization were part of model development, we additionally conducted a nested grouped sensitivity analysis to evaluate the robustness of these training-side choices. The outer loop again held out one complete biological replicate, and the 12 outer-test plots remained inaccessible during preprocessing, parameter selection, and training. Within each 24-plot outer training set, the other two biological replicates alternated as inner validation folds. Candidate configurations were selected by RMSE calculated from the pooled inner predictions. The candidate current-node weights for endpoint-aware pooling were α ∈ {0, 0.15, 0.30, 0.45}; the complementary weight was assigned to the global-mean representation. Two combinations of dropout, weight decay, and learning rate were evaluated: 0.15/0.04/0.007 and 0.25/0.08/0.006, each trained for 420 epochs. All three outer training sets selected α = 0.45 and the stronger regularization configuration. Each selected model was then refitted on its complete outer training set and used to predict the held-out outer replicate. This analysis served as a sensitivity check on the early-grain-filling main result and on the robustness of training-side configuration selection (Varma and Simon, 2006). The feature set and the early-grain-filling cutoff were retained from the primary analysis so that this sensitivity analysis specifically assessed the robustness of endpoint-pooling and regularization selection. It was therefore not intended to constitute fully nested re-selection of the feature set, model family, or cutoff stage.

Variable contributions were evaluated by grouped, fold-specific trajectory permutation. Within each held-out biological replicate, the complete trajectory of one variable and its corresponding differences were jointly permuted among plots 30 times while temporal order was preserved. Importance was defined as the increase in RMSE relative to the original held-out prediction. This measure describes the fitted model’s dependence on a variable trajectory. Because correlated variables can share information, the ranking was not interpreted as an independent biological effect or a causal relationship.

Model performance was evaluated using R^2^, RMSE, MAE, and bias. Bias was defined as the mean difference between predicted and observed yield (predicted − observed); positive values indicate overall overprediction and negative values indicate underprediction. Overall metrics were recalculated after pooling grouped out-of-fold predictions from all 36 plots. Fold-specific R^2^ values were calculated from the 12 plots in each held-out replicate, and the standard deviation across the three folds described performance variation among biological replicates. Because overall and fold-specific R^2^ use different total sums of squares, the overall value is not the arithmetic mean of the three fold-specific values. Where paired bootstrap intervals are reported, plot-level predictions were resampled with replacement within biological replicate, the same sampled plot indices were applied to both members of a comparison, and the 2.5th and 97.5th percentiles of 10,000 resamples defined the 95% confidence interval. These paired intervals quantify conditional uncertainty after the compared prediction pipelines are fixed and do not incorporate uncertainty from all preceding model-development choices. The evaluation metrics are defined in (Equations 10-13).

(10) $$R^{2}=1-\frac{\sum {\left(y_{i}-{\hat{y}}_{i}\right)}^{2}}{\sum {\left(y_{i}-\bar{y}\right)}^{2}}$$

(11) $$\text{RMSE}=\sqrt{\frac{1}{n}\sum {\left(y_{i}-{\hat{y}}_{i}\right)}^{2}}$$

(12) $$\text{MAE}=\frac{1}{n}\sum |y_{i}-{\hat{y}}_{i}|$$

(13) $$\text{Bias}=\frac{1}{n}\sum ({\hat{y}}_{i}-y_{i})$$

Multispectral image processing and vegetation-index calculation were performed in Python 3.13.5 using OpenCV 4.12.0, Rasterio 1.4.3, NumPy 2.2.6, and pandas 2.2.3. Point-cloud preprocessing, extraction of three-dimensional structural variables, and visualization were performed mainly with Open3D 0.19.0. Correlation analysis and conventional machine learning used SciPy 1.16.3 and scikit-learn 1.6.1. Temporal deep-learning models were implemented in PyTorch 2.12.1, and figures were prepared with Matplotlib 3.10.0.

## 3 Results

### 3.1 Measurement agreement and modality-specific trait responses

Point-cloud-derived plant height agreed closely with manual measurements across the nine common growth stages, with stage-specific R^2^ ranging from 0.912 to 0.976 and RMSE from 0.78 to 2.21 cm (**Figure 5**). LAI-2200C also agreed closely with manual LAI measurements (R^2^ = 0.948; RMSE = 0.417; **Figure 6**), supporting these measurements as reference phenotypes for characterizing sensor response.

**Figure 5:**
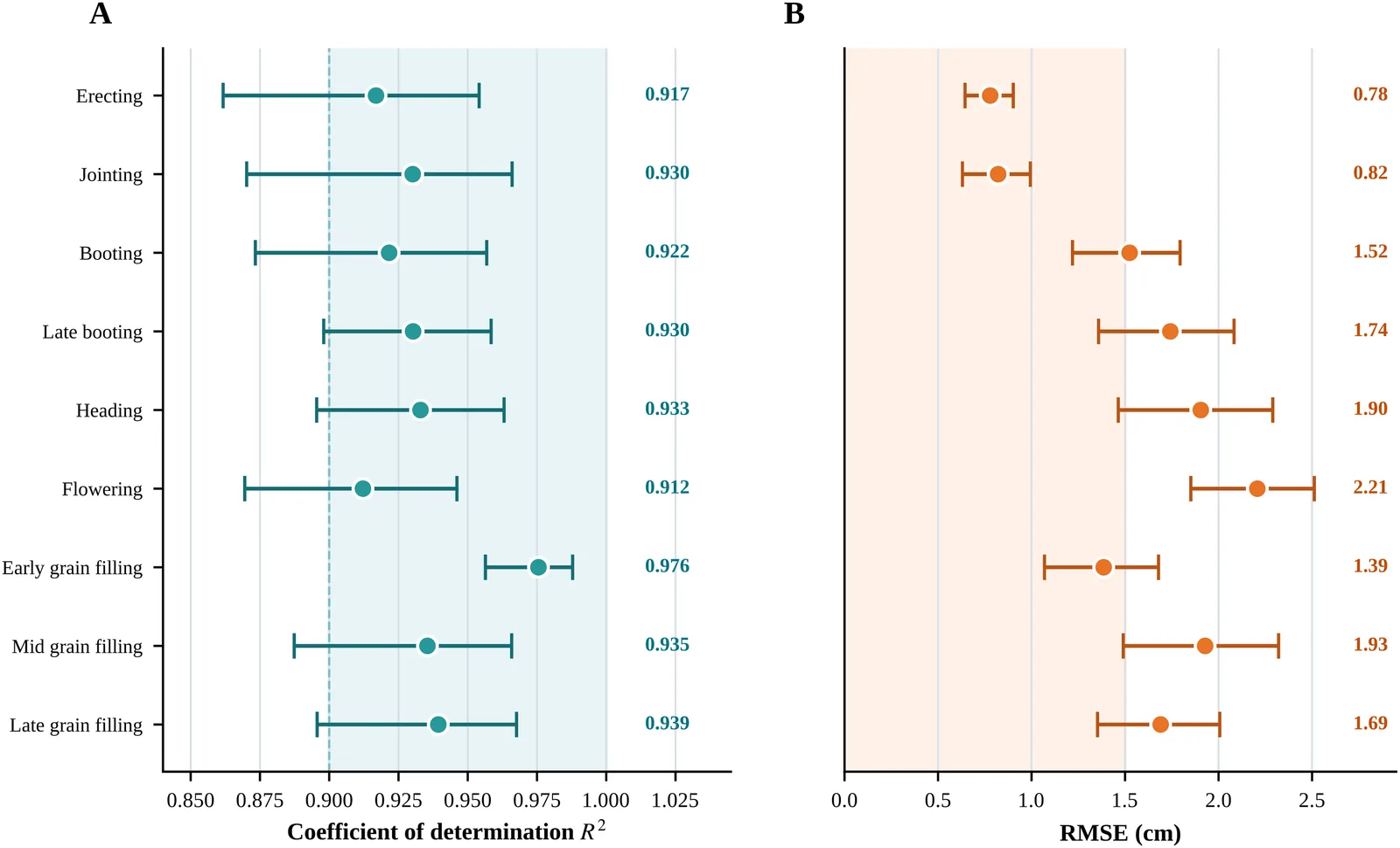
Stagewise validation of point-cloud-derived plant height. **(A)** R^2^ between point-cloud-derived H99.5 and manually measured plant height at each growth stage. **(B)** Corresponding RMSE. Error bars indicate 95% bootstrap confidence intervals; n = 36 plots at each stage.

**Figure 6:**
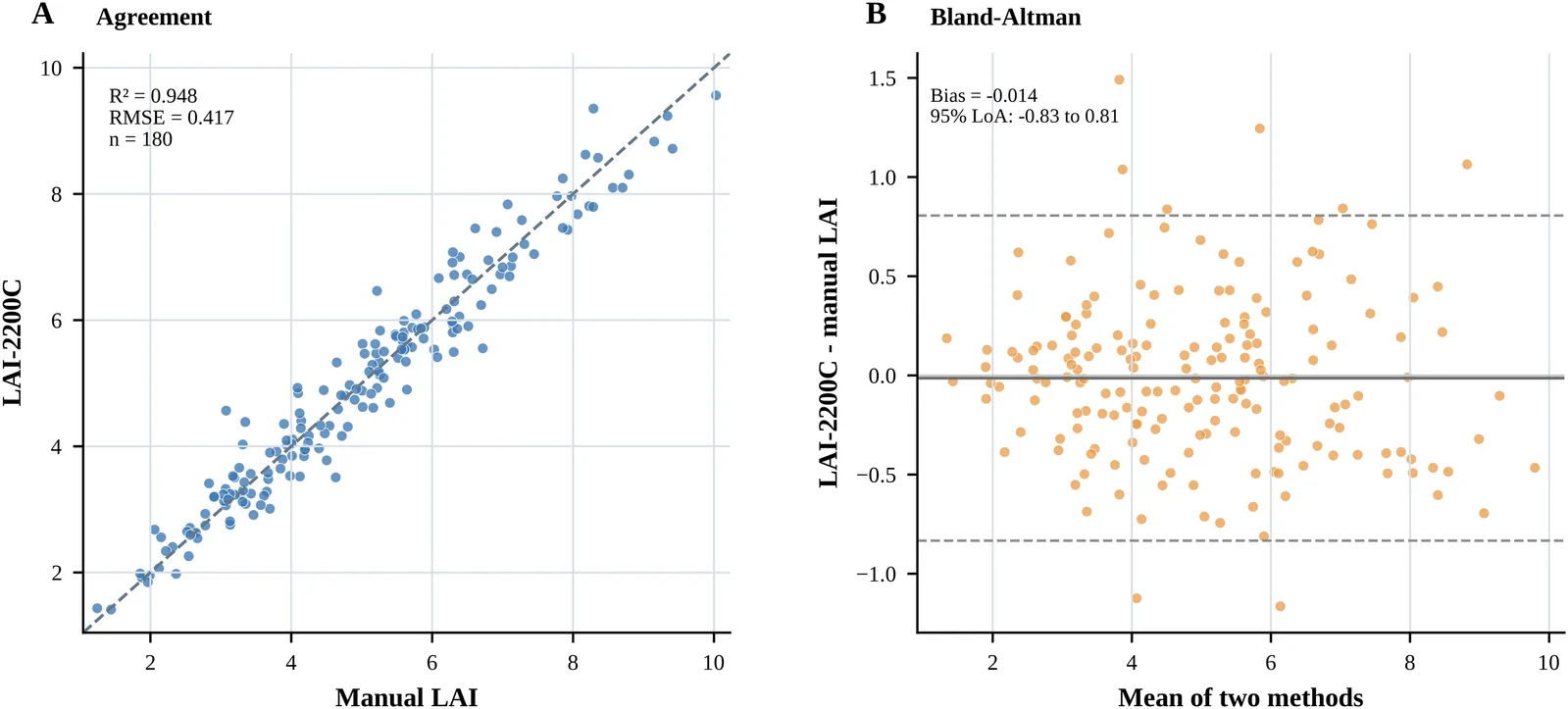
Agreement between LAI-2200C and manual LAI measurements. **(A)** The 180 paired observations and the 1:1 line. **(B)** Bland–Altman differences, mean bias, and 95% limits of agreement.

For SPAD, the 14-stage multispectral ExtraTrees model produced the highest internal cross-validation point estimate among the evaluated configurations (R^2^ = 0.914; RMSE = 2.830; MAE = 2.052), compared with R^2^ = 0.864, 0.895, and 0.910 for stage-specific multispectral, point-cloud, and fused models, respectively (**Figure 7**; **Table 4**). The result indicates that repeated spectral observations captured seasonal SPAD variation more completely than single-stage inputs.

**Figure 7:**
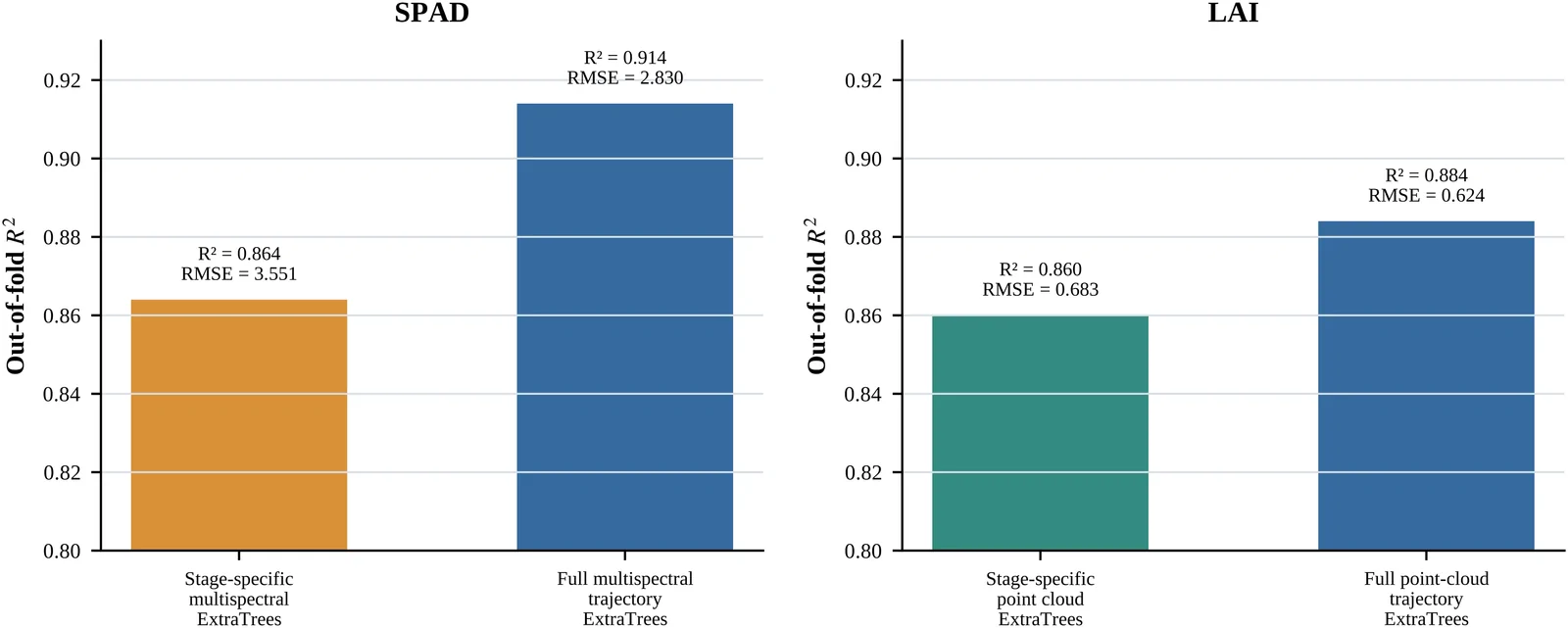
Stage-specific and full-season trajectory estimation of SPAD and LAI. Bar heights indicate R^2^ calculated from 324 stage-level out-of-fold predictions. Stage-specific models used input from the target stage, whereas full-season trajectory models used either the 14-stage multispectral trajectory or the 10-stage point-cloud trajectory from each of the 36 plots to estimate trait values at the nine common growth stages. Labels report R^2^ and RMSE.

**Table 4:** Performance of plant-height measurement and stage-specific and full-season trajectory estimation of SPAD and LAI.

Target	Data organization	Data source	Model or metric	R^2^	RMSE	MAE
Plant height	Direct stage-specific measurement	Point cloud	H99.5	0.912–0.976	0.78–2.21 cm	0.61–1.83 cm
SPAD	Stage-specific	Multispectral	ExtraTrees	0.864	3.551	2.627
SPAD	Complete 14-stage trajectory	Multispectral	ExtraTrees	0.914	2.830	2.052
LAI	Stage-specific	Point cloud	ExtraTrees	0.860	0.683	0.526
LAI	Complete 10-stage trajectory	Point cloud	ExtraTrees	0.884	0.624	0.486

Plant height is reported in centimeters; SPAD is expressed in SPAD units; LAI is dimensionless. The plant-height row reports the range across nine growth stages.

For LAI, the 10-stage point-cloud ExtraTrees trajectory yielded R^2^ = 0.884, RMSE = 0.624, and MAE = 0.486, compared with R^2^ = 0.860 for the stage-specific point-cloud model and 0.770 and 0.847 for stage-specific multispectral and fused models, respectively (**Figure 7**; **Table 4**). These phenotype analyses establish the sensing basis for the subsequent yield-prediction analysis rather than serving as independent prediction objectives.

### 3.2 Correlation structure and low-redundancy variable selection

The 15 vegetation indices contained several highly redundant pairs (**Figure 8A**). Spearman’s ρ reached 0.999 for EVI2 and MSAVI, 0.998 for NDRE and CIrededge, and 0.988 for NDVI and RVI. We ran minimum redundancy-maximum relevance (mRMR) separately in each 24-plot training fold. VARI ranked second in all three folds; NDRE ranked first, first, and sixth; and EVI ranked third, sixth, and third. Their mean ranks were 2.00, 2.67, and 4.00, which placed them first, second, and third in the aggregated ranking. Across the full 14-stage dataset, pairwise ρ values among VARI, EVI, and NDRE were 0.232, −0.197, and 0.099 (|ρ| ≤ 0.232).

**Figure 8:**
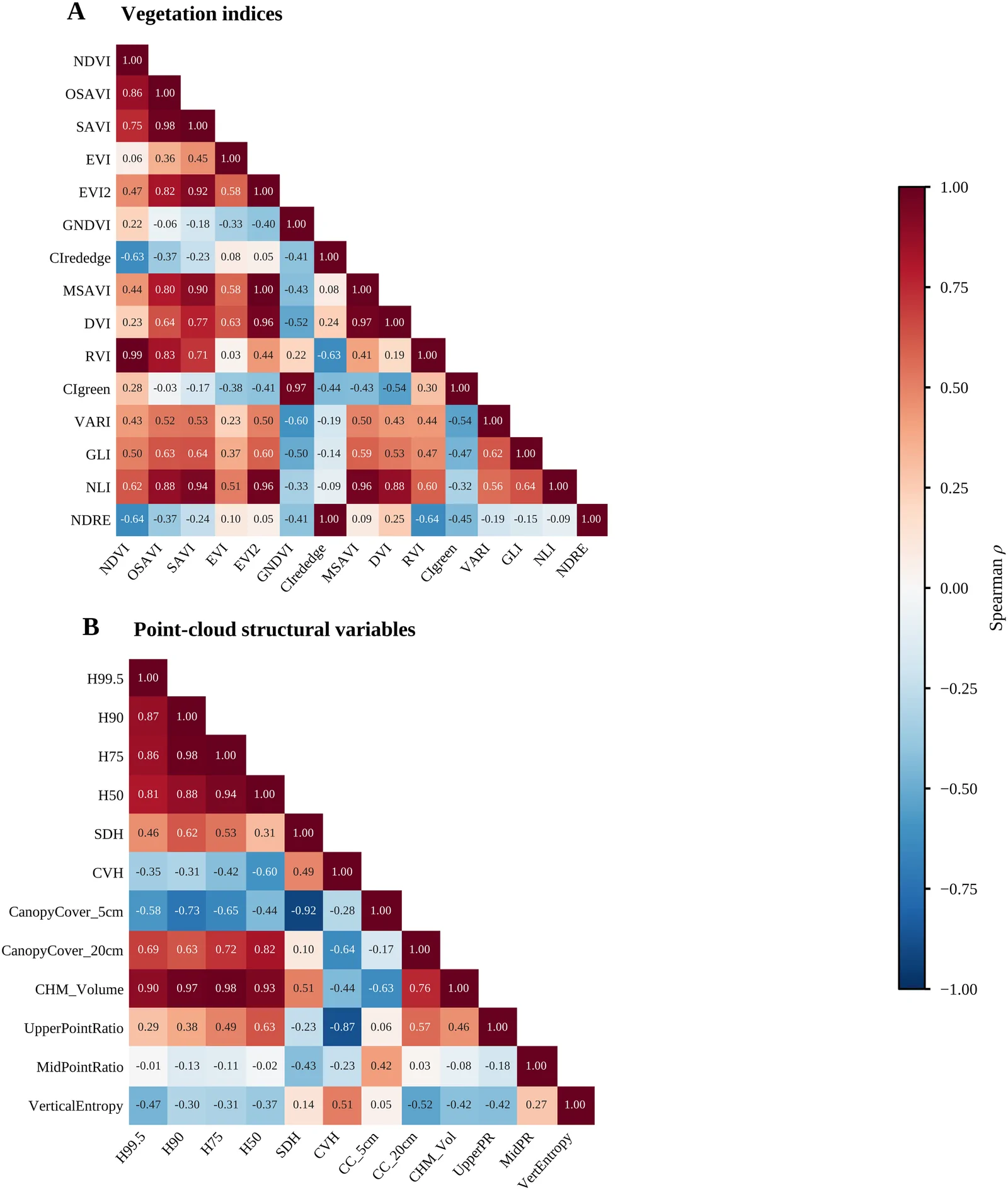
Spearman correlation structure of vegetation indices and point-cloud structural variables. **(A)** Correlation matrix for 15 vegetation indices across 14 growth stages (n = 504 plot-stage observations). **(B)** Correlation matrix for 12 point-cloud structural variables across 10 growth stages (n = 360 plot-stage observations). Both the color scale and cell labels represent Spearman’s ρ; cell values are rounded to two decimal places.

The 12 point-cloud structural variables also contained strong redundancy (**Figure 8B**). Spearman’s ρ reached 0.983 for H75 and CHM_Volume, 0.978 for H90 and H75, and 0.970 for H90 and CHM_Volume. The standard deviation of point height and CanopyCover_5cm showed a strong negative correlation (ρ = −0.916). After we centered each variable within growth stage, H75 and CHM_Volume remained closely correlated (ρ = 0.927).

Training-fold rankings, pairwise correlations, and input-size comparisons supported a compact set of three vegetation indices and seven point-cloud structural variables. VARI, NDRE, and EVI occupied the top three mean mRMR ranks and showed low pairwise correlations. Adding more indices increased the input dimension without improving yield prediction. Removing highly redundant point-cloud structural variables retained the major dimensions of canopy height, variation, cover, volume, vertical distribution, and structural complexity.

### 3.3 Yield-model comparison and multisource fusion

Temporal-GCN gave the highest R^2^ point estimate and the lowest RMSE among the five candidate yield models under matched inputs and partitions (**Table 5**). All models used the same three vegetation indices and seven point-cloud structural variables, the green-up-to-early-grain-filling window, and three-fold grouping by complete biological replicate. Temporal-GCN yielded R^2^ = 0.903, RMSE = 25.52 kg mu^-1^, and MAE = 20.84 kg mu^-1^ (1 mu = 1/15 ha). LSTM yielded 0.883, 28.09 kg mu^-1^, and 22.31 kg mu^-1^, whereas TCN yielded 0.870, 29.58 kg mu^-1^, and 23.77 kg mu^-1^. MS-PLS yielded 0.794, 37.22 kg mu^-1^, and 26.87 kg mu^-1^, and temporal SVR yielded 0.770, 39.36 kg mu^-1^, and 32.18 kg mu^-1^. Bias across the five models ranged from −5.43 to +2.76 kg mu^-1^ and showed no strong overall directional shift. The three nonlinear temporal encoders all produced higher R^2^ and lower error point estimates than the two flattened traditional baselines under these conditions. The five-model analysis was a benchmark comparison under fixed candidate configurations and common grouped partitions. Temporal-GCN yielded the highest point estimate and was then carried forward as the common framework for subsequent data-source, input-size, and stagewise analyses; no model-family re-selection was performed within individual cutoff stages.

**Table 5:** Comparison of yield-prediction models, data sources, and input-set sizes.

Comparison	Configuration/model	R^2^	RMSE (kg mu^-1^)	MAE (kg mu^-1^)	Bias (kg mu^-1^)	Description
Model architecture	Temporal-GCN	0.903	25.52	20.84	+2.76	Highest R^2^ point estimate and lowest RMSE
Model architecture	TCN	0.870	29.58	23.77	+0.96	Two-layer one-dimensional convolutional temporal encoder
Model architecture	LSTM	0.883	28.09	22.31	+2.64	Single-layer LSTM temporal encoder
Model architecture	MS-PLS	0.794	37.22	26.87	−5.43	Unfolded conventional baseline
Model architecture	Temporal SVR	0.770	39.36	32.18	−1.20	Unfolded RBF-SVR baseline
Data source	Fusion: 3 VI + 7 PC	0.903	25.52	20.84	+2.76	Equal-condition fusion model
Data source	Point cloud: 7 PC	0.898	26.21	22.50	+1.00	Structural single-source baseline
Data source	Multispectral: 3 VI	0.828	33.99	29.58	−1.28	Spectral single-source baseline
VI-set size	Fusion: 4 VI + 7 PC	0.886	27.75	23.29	−0.64	mRMR expansion: CIgreen added
VI-set size	Fusion: 8 VI + 7 PC	0.884	27.96	21.97	+1.06	Four additional high-ranking VIs added
VI-set size	Fusion: 15 VI + 7 PC	0.883	28.08	23.32	+1.42	All candidate VIs

VI and PC denote vegetation index and point-cloud structural variable, respectively. In the model-architecture comparison, all five candidate models used the same 3 VI + 7 PC inputs, the green-up-to-early-grain-filling window, and the same three-fold partition by complete biological replicate. MS-PLS and temporal SVR modeled the same unfolded 8 × 22 temporal tensor; TCN, LSTM, and Temporal-GCN encoded node-level temporal inputs directly. In the data-source and VI-set comparisons, the final Temporal-GCN was fixed. The 4-, 8-, and 15-VI configurations progressively expanded the feature set according to mean mRMR rank across the three training folds. Single-source routes retained only the corresponding sensor-availability mask, whereas the fused route retained both masks. Bias = mean(predicted − observed); positive values indicate overall overprediction and negative values indicate underprediction. All temporal inputs included state values and signed differences between adjacent stages; structural nonacquisition was represented by sensor-availability masks. One mu equals 1/15 ha.

Fusion yielded the highest R^2^ and the lowest RMSE and MAE in the equal-condition data-source comparison (**Figure 9**; **Table 5**). The point-cloud model yielded R^2^ = 0.898, RMSE = 26.21 kg mu^-1^, MAE = 22.50 kg mu^-1^, and bias = +1.00 kg mu^-1^. The multispectral model yielded R^2^ = 0.828, RMSE = 33.99 kg mu^-1^, MAE = 29.58 kg mu^-1^, and bias = −1.28 kg mu^-1^. Combining three vegetation indices with seven point-cloud structural variables yielded R^2^ = 0.903, RMSE = 25.52 kg mu^-1^, MAE = 20.84 kg mu^-1^, and bias = +2.76 kg mu^-1^. The fusion model yielded fold-specific R^2^ values of 0.908, 0.925, and 0.876, with a fold-to-fold standard deviation of 0.025. The point-cloud model had a fold-to-fold standard deviation of 0.048. Pooling all 36 out-of-fold predictions yielded an overall R^2^ distinct from the arithmetic mean of the fold-specific values. Paired bootstrap analysis gave ΔR^2^ = 0.005, with a 95% confidence interval (CI) from −0.028 to 0.037. The corresponding RMSE reduction was 0.69 kg mu^-1^ (95% CI, −3.33 to 4.23 kg mu^-1^).

**Figure 9:**
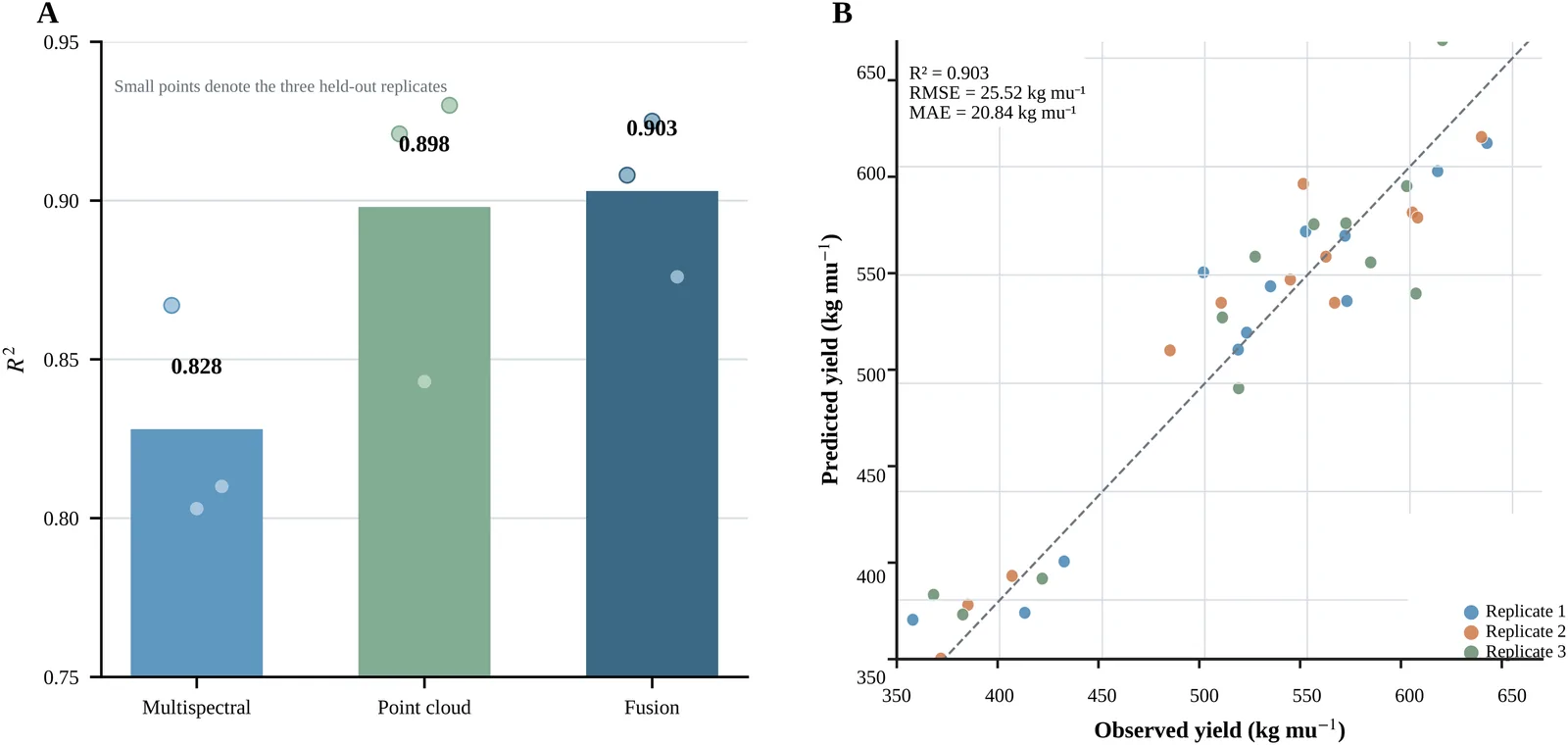
Yield-prediction performance of the final Temporal-GCN. **(A)** Overall R^2^ calculated from the pooled out-of-fold predictions and fold-specific R^2^ values for the three held-out biological replicates under the multispectral, point-cloud, and fused routes. **(B)** Held-out predictions for all 36 plots from the fused model combining three vegetation indices and seven point-cloud structural variables. The dashed line is the 1:1 line, and colors distinguish the three biological replicates.

Every expanded vegetation-index set yielded lower performance than the three-index fusion model. The 3-, 4-, 8-, and 15-index configurations yielded R^2^ values of 0.903, 0.886, 0.884, and 0.883, respectively. Their RMSE values were 25.52, 27.75, 27.96, and 28.08 kg mu^-1^. Their bias values were +2.76, −0.64, +1.06, and +1.42 kg mu^-1^. A nested grouped sensitivity analysis selected endpoint-pooling and regularization settings exclusively within each outer training set. The pooled outer-fold predictions yielded R^2^ = 0.896, RMSE = 26.47 kg mu^-1^, and MAE = 22.03 kg mu^-1^, with outer-fold R^2^ ranging from 0.856 to 0.921. The nested estimate remained close to the main three-fold result of R^2^ = 0.903.

### 3.4 Prediction window and variable contributions

Cumulative-to-current-stage yield-prediction performance reached its highest point estimate at early grain filling (**Figure 10A**). With the input set, Temporal-GCN, grouped partitions, and random seeds fixed, R^2^ increased from 0.607 at erecting to 0.756 at jointing and 0.792 at booting. R^2^ declined to 0.724 at late booting, increased to 0.883 at heading, and reached 0.862 at flowering. The early-grain-filling cutoff yielded R^2^ = 0.903, RMSE = 25.52 kg mu^-1^, and MAE = 20.84 kg mu^-1^. Adding mid-grain-filling, late-grain-filling, and maturity observations yielded R^2^ values of 0.900, 0.852, and 0.867. None of these later cutoffs exceeded the early-grain-filling point estimate. This stagewise maximum arose from the same full-season cutoff analysis and is therefore interpreted as the highest within-dataset grouped-cross-validation point estimate among the evaluated cutoffs, rather than as an independently validated universal optimum.

**Figure 10:**
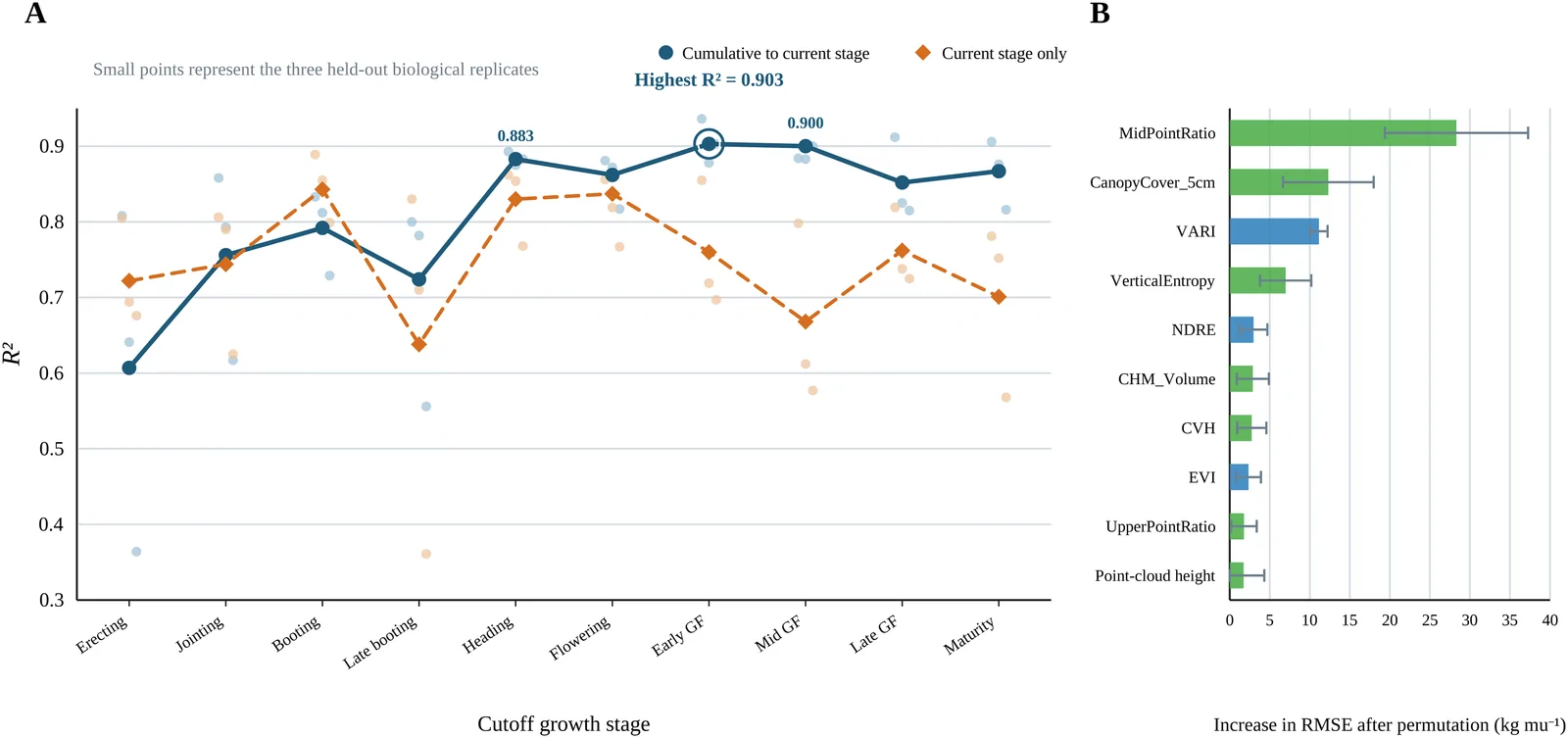
Stagewise cutoff performance and variable contributions to yield prediction. **(A)** Overall and fold-specific R^2^ values for current-stage-only and cumulative-to-current-stage inputs from erecting through maturity using the final Temporal-GCN. Large points denote the overall R^2^ calculated from 36 pooled out-of-fold predictions, and small points denote fold-specific results for the three held-out biological replicates. Early grain filling yielded the highest overall R^2^ point estimate. **(B)** Trajectory-permutation importance for the cumulative early-grain-filling model. Bars show the mean increase in RMSE after permutation; error bars show the standard deviation across the three held-out biological replicates.

The R^2^ difference between cumulative-to-current-stage and current-stage-only inputs varied by growth stage. At booting, the current-stage-only model yielded R^2^ = 0.845, compared with 0.792 for the cumulative-to-current-stage model. At heading, the cumulative-to-current-stage model yielded R^2^ = 0.883, compared with 0.831 for the current-stage-only model. The largest R^2^ difference occurred at early grain filling, where the respective values were 0.903 and 0.760. Paired bootstrap resampling of the 36 held-out plot-level predictions within biological replicate gave ΔR^2^ = 0.143 (95% CI, 0.059 to 0.299) for cumulative versus current-stage-only inputs at early grain filling, with an RMSE reduction of 14.69 kg mu^-1^ (95% CI, 7.28 to 22.02 kg mu^-1^). The paired difference between early and mid-grain filling was ΔR^2^ = 0.003 (95% CI, −0.015 to 0.025). Early grain filling therefore represents the highest point estimate in the full-season analysis; the small difference from mid grain filling was not statistically distinguishable based on the paired interval.

Trajectory-permutation importance concentrated on canopy structure at the early-grain-filling cutoff (**Figure 10B**). Permuting MidPointRatio increased RMSE by 28.32 kg mu^-1^. CanopyCover_5cm and VARI increased RMSE by 12.31 and 11.14 kg mu^-1^, and VerticalEntropy increased it by 6.99 kg mu^-1^. The corresponding increases for NDRE, CHM_Volume, CVH, EVI, UpperPointRatio, and point-cloud height were 2.97, 2.89, 2.75, 2.34, 1.78, and 1.73 kg mu^-1^. Removing MidPointRatio while holding the remaining inputs and training scheme fixed reduced R^2^ from 0.903 to 0.872 and increased RMSE from 25.52 to 29.33 kg mu^-1^. The agreement between permutation and ablation results showed that predictions were especially sensitive to MidPointRatio within the present pipeline.

## 4 Discussion

### 4.1 Trait responses to structural and spectral information

The modality-specific trait results followed the measurement principles of LiDAR and multispectral imaging. LiDAR directly records three-dimensional canopy geometry and responds to plant height, cover, and vertical organization. Previous studies have used LiDAR to quantify wheat height, ground cover, aboveground biomass, and effective leaf area index (Jimenez-Berni et al., 2018; Yang et al., 2023; Mulero et al., 2025). The stagewise agreement reported in Section 3.1 supports H99.5 as a consistent plot-level height measure across the observed stages. The point-cloud trajectory also gave the highest LAI-estimation point estimate among the evaluated full-season configurations. Because the temporal organizations differed, these trait analyses were used primarily to characterize modality-specific responses rather than to rank the intrinsic performance of the two sensors.

Multispectral trajectories captured seasonal variation in canopy greenness more accurately than single-stage observations. Visible, red-edge, and near-infrared reflectance respond to chlorophyll content, nitrogen status, and canopy senescence (Gitelson et al., 2005; Hassan et al., 2018; Jiang et al., 2022). The full-season result in Section 3.1 indicates that repeated spectral observations represented leaf-greenness development more accurately than the stage-specific alternatives. The relative performance of each modality depended on the match between its measurement principle and the biophysical basis of the target trait.

The correlation analysis showed that several candidate variables encoded overlapping canopy information. H75, H90, and CHM_Volume all represented canopy height or volume, whereas NDRE and CIrededge both represented red-edge responses. The mRMR criterion balanced target relevance against redundancy (Section 2.3; Peng et al., 2005) and ranked VARI, NDRE, and EVI highest on average across the three training folds. Together, the three retained indices represented visible-band greenness, relative canopy spectral response, and red-edge status. The point-cloud screening likewise removed redundant height and volume variables while preserving the major structural dimensions. The persistent association between H75 and CHM_Volume after within-stage centering indicated plot-level covariation in canopy height and volume beyond their shared seasonal trend.

The trait responses and correlation structure provide a biophysical basis for multisource yield prediction. Point-cloud trajectories track canopy establishment, spatial expansion, and vertical development. Multispectral trajectories add information about greenness, red-edge response, and senescence. Previous wheat studies link fusion benefits to preserved complementary signals and identify input count alone as an unreliable basis for improvement (Li et al., 2022; Gu et al., 2024). The compact input set retained the main response dimensions of both sensors under a common temporal model.

### 4.2 Prediction window for pre-maturity yield estimation

The stagewise cutoff analysis identified early grain filling as the highest R^2^ point estimate (Section 3.4), while performance at the adjacent mid-grain-filling cutoff was similarly high. The nested grouped sensitivity analysis also retained high early-grain-filling performance when endpoint-pooling and regularization were selected entirely within the outer training sets. Together, these results identify early grain filling as the strongest candidate prediction window in this dataset. This cutoff should be interpreted as the highest-performing window among those evaluated within the present grouped-cross-validation framework, rather than as an independently validated universal optimum.

The contrast between cumulative-to-current-stage and current-stage-only inputs varied across growth stages (**Figure 10A**). The largest difference occurred at early grain filling (R^2^ = 0.903 versus 0.760), substantially exceeding the differences observed between sensor combinations and adjacent cutoff windows. Paired bootstrap resampling supported this within-experiment contrast (ΔR^2^ = 0.143; 95% CI, 0.059 to 0.299), with an RMSE reduction of 14.69 kg mu^-1^ (95% CI, 7.28 to 22.02 kg mu^-1^). This makes the incremental information carried by the accumulated trajectory one of the clearest predictive signals in the study. The result supports the conclusion that multitemporal observations provide yield-relevant information beyond a single-stage measurement during reproductive development. Because the cumulative model also receives more observations and uses the Temporal-GCN representation, the gain should not be attributed solely to the graph architecture.

The cutoff trajectory was nonmonotonic, with local declines at late booting and flowering, a modest early-to-mid-grain-filling change, and larger declines at the final stages. Previous multitemporal wheat-yield studies likewise report that growth stages differ in the yield information they provide (Tanabe et al., 2023). We therefore emphasize robust stage-to-stage patterns and the highest point estimate when interpreting the cutoff trajectory.

The early-grain-filling cutoff retained about 27 d of lead time before harvest. Heading occurred on 29 April 2026, about 40 d before harvest, and early grain filling occurred on 12 May 2026. The three subsequent acquisitions on 21 May, 28 May, and 4 June all yielded lower overall R^2^ values. Early grain filling therefore offers a candidate sensing window that balances prediction accuracy with acquisition timing.

### 4.3 Fusion gains and scope of applicability

The compact configuration of three vegetation indices and seven point-cloud structural variables retained complementary spectral and structural information. MidPointRatio, CanopyCover_5cm, and VerticalEntropy described canopy organization. VARI, EVI, and NDRE represented visible-band greenness, relative canopy spectral response, and red-edge status. The input-size results link diversity to complementary response dimensions and identify variable count alone as an unreliable route to improvement (Li et al., 2022; Gu et al., 2024).

Fusion produced the highest point estimate under matched conditions. Because the paired 95% intervals for ΔR^2^ and RMSE reduction crossed zero (Section 3.3), we emphasize the complementary structural–spectral information captured by fusion rather than a statistically significant difference from LiDAR alone. The model comparison likewise showed that Temporal-GCN ranked highest among the five candidate models; given the relatively small differences from LSTM and TCN, this provides evidence of a within-dataset model ranking. The contribution of the framework thus rests on controlled integration of multitemporal structural and spectral information and on identifying when that information becomes useful for yield prediction.

The study design defines the external scope of these estimates rather than the validity of the within-experiment comparisons. The experiment included three cultivars, four nitrogen rates, three biological replicates, one site, one growing season, and 36 plots. Grouped cross-validation kept every plot’s temporal records within one partition and reduced the risk of temporal information leakage (Section 2.4; Roberts et al., 2017). The resulting estimates quantify stability across biological replicates within the observed cultivar-by-nitrogen combinations, while broader transferability is appropriately addressed through future independent validation. The strip arrangement linked nitrogen treatment with field position, while spatial position was not included as a model predictor. Accordingly, the present analysis focuses on predictive performance rather than causal partitioning of nitrogen and spatial effects. Importantly, these design considerations do not alter the central within-experiment finding that multitemporal observations improved yield discrimination over single-stage inputs during reproductive development.

Our acquisition and preprocessing pipeline fixed sensor height, scanning geometry, and downsampling settings (Section 2.2), providing a consistent basis for interpreting variable importance within the present workflow. Under this configuration, MidPointRatio showed the largest contribution and emerged as the strongest model-associated structural predictor. Cross-platform comparisons should harmonize scanning geometry, point density, and downsampling procedures.

## 5 Conclusion

This study aligned ground-based LiDAR canopy structure with multispectral canopy responses on a common phenological axis. In grouped three-fold cross-validation, the fused Temporal-GCN reached its highest full-season cutoff point estimate at early grain filling (R^2^ = 0.903; RMSE = 25.52 kg mu^-1^; MAE = 20.84 kg mu^-1^), approximately 27 d before harvest. The cumulative-to-current-stage model showed its largest R^2^ difference from the current-stage-only model at this stage (0.903 versus 0.760; paired ΔR^2^ = 0.143, 95% CI, 0.059 to 0.299), with paired uncertainty supporting the additional yield-relevant information provided by historical observations during reproductive development. Performance remained similarly high at mid grain filling, while fusion integrated complementary structural and spectral information and achieved the highest point estimate under matched conditions. Nested grouped sensitivity analysis retained high early-grain-filling performance when endpoint-pooling and regularization settings were selected within the outer training sets. Overall, the framework provides a practical basis for identifying when multitemporal structural and spectral observations become sufficiently informative for pre-maturity winter wheat yield prediction.

## Data Availability

The raw data supporting the conclusions of this article will be made available by the authors, without undue reservation.
